# A distinct M2 macrophage infiltrate and transcriptomic profile decisively influence adipocyte differentiation in lipedema

**DOI:** 10.3389/fimmu.2022.1004609

**Published:** 2022-12-20

**Authors:** Stefan Wolf, Jenna H. Rannikko, Reetta Virtakoivu, Paolo Cinelli, Gunther Felmerer, Anna Burger, Pietro Giovanoli, Michael Detmar, Nicole Lindenblatt, Maija Hollmén, Epameinondas Gousopoulos

**Affiliations:** ^1^ Department of Plastic Surgery and Hand Surgery, University Hospital Zurich, Zurich, Switzerland; ^2^ MediCity Research Laboratory, University of Turku, Turku, Finland; ^3^ Department of Trauma Surgery, University Hospital Zurich, Zurich, Switzerland; ^4^ Division of Plastic Surgery, Department of Trauma Surgery, Orthopedics and Plastic Surgery, University Medical Center Göttingen, Georg-August-University, Göttingen, Germany; ^5^ Institute of Pharmaceutical Sciences, Swiss Federal Institute of Technology, ETH Zurich, Zurich, Switzerland

**Keywords:** lipedema, macrophage, CD163, adipogenic differentiation, adipose tissue, CyTOF

## Abstract

Lipedema is a chronic and progressive adipose tissue disorder, characterized by the painful and disproportionate increase of the subcutaneous fat in the lower and/or upper extremities. While distinct immune cell infiltration is a known hallmark of the disease, its role in the onset and development of lipedema remains unclear. To analyze the macrophage composition and involved signaling pathways, anatomically matched lipedema and control tissue samples were collected intra-operatively from gender- and BMI-matched patients, and the Stromal Vascular Fraction (SVF) was used for Cytometry by Time-of-Flight (CyTOF) and RNA sequencing. The phenotypic characterization of the immune component of lipedema versus control SVF using CyTOF revealed significantly increased numbers of CD163 macrophages. To gain further insight into this macrophage composition and molecular pathways, RNA sequencing of isolated CD11b+ cells was performed. The analysis suggested a significant modification of distinct gene ontology clusters in lipedema, including cytokine-mediated signaling activity, interleukin-1 receptor activity, extracellular matrix organization, and regulation of androgen receptor signaling. As distinct macrophage populations are known to affect adipose tissue differentiation and metabolism, we evaluated the effect of M2 to M1 macrophage polarization in lipedema using the selective PI3Kγ inhibitor IPI-549. Surprisingly, the differentiation of adipose tissue-derived stem cells with conditioned medium from IPI-549 treated SVF resulted in a significant decreased accumulation of lipids in lipedema versus control SVF. In conclusion, our results indicate that CD163+ macrophages are a critical component in lipedema and re-polarization of lipedema macrophages can normalize the differentiation of adipose-derived stem cells *in vitro* evaluated by the cellular lipid accumulation. These data open a new chapter in understanding lipedema pathophysiology and may indicate potential treatment options.

## 1 Introduction

Lipedema is an adipose tissue disorder characterized by a symmetrical bilateral increase of painful subcutaneous fat tissue, predominantly in the legs and arms. This medical condition is a significant burden for the individual patient causing pain, considerable disability, and psychological distress (1). Lipedema occurs almost exclusively in women, and the disease shows frequently a familial clustering. The accumulation of fibro-adipose tissue is associated with hormonal changes, such as puberty, pregnancy, or menopause. The volume increase can be attributed to both, the proliferation of adipose stem cells and adipocyte hypertrophy (2), which leads to the distinct lipedema phenotype and the typical adipose tissue distribution. Multiple studies have revealed that adipose-derived stem cells from lipedema patients have a higher adipogenic differentiation potential (3, 4).

The volume increase in the extremities is often misdiagnosed as other clinical entities, such as obesity and lymphedema. All these entities are characterized by a distinct but different immune cell infiltrate. Recent research from various independent groups including ours has identified an increased infiltration of macrophages in lipedema tissue, while the T cell compartment remains unaltered. Interestingly, the infiltrating macrophages exhibit an M2 polarization state, attributed to the increased expression of CD163 (5). By contrast, adiposity is normally characterized by a predominance of M1 macrophages (6), and lymphedema by the infiltration of CD4+ T cells (7). Although CD4+ cell recruitment regulates fibrosis formation in lymphedema (8) the function of macrophages is less clear in lipedema. It is well established that macrophages, can influence adipogenic differentiation and adipocyte metabolism *via* cytokine secretion. Recent studies have observed altered systemic cytokine levels in lipedema patients, possibly attributed to the infiltrating macrophages, as well as an increased metabolic activity of the stromal vascular fraction (SVF) of the affected lipedematous extremities (9). The metabolism of the adipose tissue is mostly determined by the interplay between adipocytes, preadipocytes, endothelial cells and immune cells. In lipedema, both the SVF cell composition and the cell interplay appears altered, which seems to be a potential target in the treatment of lipedema.

In the current study, we performed a detailed phenotypic analysis of the myeloid component of the stromal vascular fraction of lipedema patients and anatomical site and BMI matched controls, to analyze in depth the predominant macrophage infiltrate. Furthermore, in an attempt to gain further insights into the macrophage transcriptomic profile and the molecular pathways involved, we performed RNA sequencing of isolated CD11b+ cells. The targets identified using the aforementioned techniques were further assessed in functional *in vitro* assays, to elucidate their contribution in the adipogenic differentiation and thus potential involvement in lipedema pathophysiology.

## 2 Materials and methods

### 2.1 Study population

The study protocols were approved by the Swiss ethics (BASEC-Nr.: 2019-00389) and Ethical Committee of the University Hospital Goettingen, State of Lower Saxony, Germany (Nr. 23-11-17, accepted on 23. November 2017). The study was conducted according to the principles of the Declaration of Helsinki. All patients were informed prior to the surgical procedures in oral and written form and provided their written informed consent. Tissue was collected from lipedema patients undergoing elective surgery of the affected extremities. As a healthy control served patients who underwent similar elective surgeries and could provide anatomical site matching fat and skin samples. Patient characteristics of the study cohort are shown in **Supplementary Table 1** and the patient characteristics for each analysis are provided in **Supplementary Table 2**.

### 2.2 Tissue collection and histology

Fat tissue and skin specimens were collected during the operating procedure and fixed in paraformaldehyde/phosphate-buffered saline at 4°C and processed further for histology. Afterwards, the skin samples were embedded in paraffin and for histological analysis of the skin tissue architecture, the specimens were cut into 5-μm thick paraffin sections. Sample preparation and staining was performed by the Center for Surgical Research of the University Hospital Zurich.

### 2.3 Immunohistochemistry

For the immunohistochemical staining, the deparaffinization of paraffin-embedded sections was followed by the rehydration of the sections. Target Retrieval Solution high was used for antigen retrieval, and endogenous peroxidase activity was blocked using 3% hydrogen peroxide (Merck). After blocking (goat serum), the sections were incubated with polyclonal rabbit antihuman CD206 (abcam ab64693; 1:2000) or monoclonal rabbit antihuman CD163 (abcam ab 182422; 1:300) antibodies. After washing with PBS, bound antibody was visualized using the DAB substrate (Chromogen), following the manufacturer’s instructions.

Histology images were obtained using a Zeiss Axio Scan Z1 equipped with a Hitachi HV-F202FCL and the whole tissue section was scanned using a Plan Apochromat 20x/0.8 numerical aperture objective.

### 2.4 RNA extraction and quantitative polymerase chain reactions

Fat tissue was collected during the operating procedure and immediately flash frozen in liquid nitrogen. RNA was isolated from a 100 mg piece of fat tissue using the RNeasy Lipid Tissue Mini Kit (Qiagen). For RNA from SVF and sorted cells the NucleoSpin RNA Plus XS kit (Macherey-Nagel) was used. Complementary DNA was transcribed from 500 ng RNA template, using the High-Capacity cDNA Reverse Transcription Kit (ThermoFisher Scientific). The polymerase chain reactions were performed using Fast SYBR Green Master Mix (Applied Biosystems) and QuantStudio 5 Real-Time PCR Systems. B2M was used as housekeeping gene, and fold changes of gene expression were calculated using the ΔΔCT method. Primer Sequences are provided in **Supplementary Table 3**.

### 2.5 Isolation of the stromal vascular fraction

For the isolation of the SVF, adipose tissue was digested using 2 mg/mL collagenase II in RPMI medium. After 1 h of incubation under moderate shaking at 37°C, cell suspension was centrifuged at 1000× *g* for 5 min. Red blood cells eliminated by incubating the cell pellet with erythrocyte lysis buffer for 10 min on ice. Subsequently, cell suspension was diluted in PBS and cells were washed with PBS and filtered through a 70 μm cell strainer. The isolated SVF cells were frozen in 45% FBS and 5% DMSO.

### 2.6 CyTOF analysis

#### 2.6.1 CyTOF antibodies and antibody labelling

List of the used antibodies including their clones, metal tags and source are provided below. The antibodies labelled in-house were obtained as carrier free (anti-human CD45) or diluted (anti-human Clever-1; clone: 9-11) in the protocol R-buffer at 200 µg/mL and used at 500 µL/labelling. For the labelling the MaxPar labelling kits (MaxPar labelling kit, Fluidigm) was used following the manufacturer’s instructions. Antibody is provided in **Table 1**.

**Table 1:** Antibody panel (grey denotes intracellular staining).

Tag	Marker	Clone	Source
144Nd	CD11b	ICRF44	Fluidigm
146Nd	CD64	10.1	Fluidigm
151Eu	CD14	M5E	Fluidigm
209Bi	CD16	3G8	Fluidigm
162Dy	CD11c	Bu15	Fluidigm
173Yb	HLA-DR	L243	Fluidigm
150Nd	CD86	IT2.2	Fluidigm
159Tb	CD274/PD-L1	29E.2A3	Fluidigm
154Sm	CD163	GHI/61	Fluidigm
171Yb	CD68	Y1/82A	Fluidigm
168Er	CD206	15-2	Fluidigm
166Er	Clever-1 (Stabilin-1)	9-11	InVivo Biotech

#### 2.6.2 CyTOF sample preparation

After thawing of the frozen SVFs cells were washed with RPMI medium. Cells were re-suspended in PBS and 0.2-1 × 10^6^ cells/sample were stained for 5 min at RT with 2.5 µM Cell-ID cisplatin viability reagent (201064; Fluidigm). For the barcoding cells were incubated for 30 min at RT with heavy-metal isotope-labelled anti-human CD45 (clone H130) antibodies (CD45_147Sm (BioLegend), 1:200), CD45_141Pr and (CD45_89Y (both Fluidigm)), washed carefully and combined. After cells were blocked with 0.2 mg/ml Kiovig for 15 min at RT the staining of the cell surface markers with heavy-metal isotope-labelled anti-human antibody cocktail was done for 30 min at RT.

For the intracellular staining the cells were permeabilized using the Transcription Factor Staining Buffer Set (00-5523-00; Invitrogen), afterwards blocked for 15min at RT with 0.2 mg/ml Kiovig and stained with heavy-metal isotope-labelled anti-human antibody cocktail for 30 min at RT. Following washing, the cells were incubated for 1 hour at RT with DNA intercalation reagent (1:1,000, Cell ID Intercalator-103Rh in MaxPar Fix and Perm Buffer; 201067; Fluidigm), washed and fixed overnight at +4 °C with 4 % paraformaldehyde solution. The following day the samples were washed, resuspended in MaxPar Water (201069; Fluidigm) containing 1:10 dilution of EQ 4 Element Beads (Fluidigm) and immediately acquired by a CyTOF mass cytometer (Helios, Fluidigm).

#### 2.6.3 CyTOF data analysis

A total of 8 healthy control samples and 7 lipedema samples were stained and analyzed by CyTOF. After excluding samples with too low cell counts (< 50 CD45^+^ cells), 5 control and 5 lipedema samples remained for analysis. Mass cytometry data was bead normalised and concatenated using CyTOF software (Fluidigm) with default settings. The.fcs files were imported into FlowJo (Treestar) and manually gated to exclude ion cloud doublets, cisplatin-positive dead cells and intercalator or event length high doublets. Debarcoding was done in FlowJo and the gated CD45^+^CD11b^+^CD64^+^ myeloid cells were exported and loaded into R (v. 4.0.4) with FlowCore package (v. 2.2.0) (10). The number of CD45^+^CD11b^+^CD64^+^ cells per sample varied from 47 to 5318 (median control group = 130, median lipedema group = 1166). For subsequent analyses, the samples were subsampled to a maximum of 1200 cells per sample, and expression values were arcsine-transformed with a cofactor of 5. Clustering and dimensionality reduction were performed based on CD11c, HLA-DR, CD86, CD163, CD206, CD14 and CD16 expression. FlowSOM (v. 1.22.0) (11) clustering was applied without further transformation or scaling in the algorithm, and a representative clustering result was chosen after running the algorithm with several seeds and number of metaclusters. Dimensionality reduction was performed with Uniform Manifold Approximation and Projection (UMAP) technique using uwot package (function UMAP, v. 0.1.10) (12), Euclidean distance as distance metric, 15 nearest neighbours in manifold approximation and 0,05 minimum distance between embedded points. For visualizing marker expression on the UMAP plots, a colour gradient was applied between 0 and 99^th^ percentile of arcsine-transformed expression values. To compare FlowSOM metaclusters, the median marker expression values in each metacluster was plotted as a heatmap using R package ComplexHeatmap (v.2.6.2, function Heatmap) (13). The relative size of each FlowSOM metacluster was calculated for each patient, and Mann-Whitney U test was used in identifying statistically significant differences between control and lipedema groups. A heatmap displaying the significance (-log_10_ *P*-value) and sign of differences in metacluster sizes between the control and lipedema group was plotted together with a dot plot showing the average metacluster sizes in control and lipedema groups as black circles with radiuses proportional to the square root of the metacluster relative size. Principal component analysis was performed based on the relative metacluster sizes of each patient (function prcomp, scaling enabled). The samples were also hierarchically clustered (Euclidean distance, complete linkage method) based on median expression levels of indicated markers on CD11b^+^CD64^+^ cells. To compare the lipedema and control group further, asinh-transformed expression values were plotted as staggered density histograms (R package ggridges, v. 0.5.3, function: geom_density_ridges). As lipedema patients had considerably more immune cells and CD11b^+^CD64^+^ cells in their SVF-samples, the above analyses were repeated with a more balanced event sampling (max. 120 cells per patient, n = 521 control group, n = 600 lipedema group) to confirm that the presented results were not caused by differences in sample sizes.

### 2.7 RNA sequencing

#### 2.7.1 Flow cytometry sorting and RNA isolation

Frozen SVFs were thawed and washed with RPMI medium. Subsequently, samples were washed with FACS Buffer (PBS with 2%FCS) and were blocked with Human TruStain FcX (Biolegend) for 15 min at RT and subsequently stained with Pacific Blue anti-human CD45 (clone HI30, Biolegend) and Brilliant Violet anti-mouse/human CD11b (M1/70, Biolegend) for 30 min at 4°C. Single-cell suspensions was washed twice with FACS buffer and were filtered through a 40-μm cell strainer. To exclude cells which died during the thawing and staining process, 5 minutes prior to the sorting Helix NP Green (Biolegend) was used for live/dead discrimination. Sorted cells were lysed and RNA was isolated using the NucleoSpin RNA Plus XS kit (Macherey-Nagel).

#### 2.7.2 Library preparation and sequencing on NovaSeq 6000

For the determination of quantity and quality of the isolated RNA a Tapestion was utilized (Agilent). The SMARTer Stranded Total RNA-Seq Kit v2 -Pico Input Mammalian (Takara Bio) was used in the succeeding steps. Briefly, 10 ng RNA per samples was reverse-transcribed using random priming into double-stranded cDNA in the presence of a template switch oligo. For the PCR amplification primers binding the random priming oligo and template switch oligo sequences, which were added to cDNA fragment during reverse transcription was used. The full-length Illumina adapters, including the index for multiplexing were added during the PCR. Ribosomal cDNA was removed by ZapR in the presence of the mammalian-specific R-Probes. Enrichment of the remaining fragment was performed with a second round of PCR amplification using Illumina adapters matching primers.

The quality and quantity of the enriched libraries were assessed with a Tapestation (Agilent, Waldbronn, German) and normalized to 10 nM in Tris-Cl 10 mM, pH8.5 supplemented with 0.1% Tween 20. Afterwards libraries were prepared following the NovaSeq workflow with the NovaSeq6000 Reagent Kit (Illumina). Cluster generation and sequencing were performed using a NovaSeq6000 System with a run configuration of single end 100bp.

#### 2.7.3 Data analysis

At first adapter sequences were removed and low-quality ends were trimmed from the raw reads. Total reads were filtered for reads with low quality (phred quality <20) using fastp (Version 0.20) (14). With the resulting high-quality reads the sequence pseudo alignment to the human reference genome (build GRCh38.p13, gene models based on Gencode release 37) was performed and quantification of gene level expression was carried out using Kallisto (Version 0.46.1) (15). For the detection of differentially expressed genes a count based negative binomial model implemented in the software package DESeq2 (R version: 4.1.2, DESeq2 version: 1.34.0) was applied (16). Genes that showed an altered expression with adjusted p-value < 0.05 (Benjamini and Hochberg method) were considered differentially expressed. Genes and samples were clustered *via* Ward’s hierarchical clustering.

### 2.8 Preparation of SVF conditioned medium

SVF were obtained as described above. 0,5 × 10^6^ thawed SVF cells were plated overnight, washed with PBS and cultured for 72 h with fresh DMEM/F12 without phenol red supplemented with or without 100nM IPI-549 at 37°C and 5% CO_2_. Medium was collected and centrifuged at 1000×*g*, 4°C for 10 min. Precipitates were discarded and supernatants were then filter sterilized and stored at −80°C.

### 2.9 Differentiation of the adipose derived stem cells

#### 2.9.1 Adipogenic differentiation of ADSCs

ADSCs were isolated from adipose tissue and were tested for the three criteria of MSCs as defined by International Society for Cellular Therapy (ISCT) in 2006 (17): Cells were plastic-adherent, expression of CD105, CD73 and CD90, and lack expression of CD45 surface molecules were tested *via* FACS and cells were able to differentiate to osteoblasts, adipocytes and chondroblasts *in vitro*. For adipogenic differentiation, ADSCs (1,5×104cells/well) were seeded into 48-well plate with DMEM complete medium. When cells were grown to confluence, medium was changed to 50% adipogenic medium (HPAd Differentiation Medium, Cell Application) and 50% SVF conditioned medium. Cells were differentiated for 7 days. The experiment was carried out in duplicates.

#### 2.9.2 Cell staining

Differentiated ADSC were washed with PBS and fixed with 4% paraformaldehyde in PBS for 30 min. After 5 min, the formaldehyde solution was exchanged. Fixed cells were washed twice with ddH_2_O and subsequently rinsed with 60% isopropanol (Sigma-Aldrich). Lipid accumulation in differentiated ADSC were stained with a 3mg/ml Oil Red O in 60% isopropanol solution (Sigma-Aldrich) for 10 min. Image acquisition was performed immediately after four ddH2O washing steps,

#### 2.9.3 Image acquisition, processing, and analysis

Image acquisition, processing, and analysis was carried out as described previously (18). Whole wells were scanned in color bright-field mode using a Cytation 5 imaging reader (BioTek). Single images were stitched together using Gen 5 image prime software (BioTek, USA, V3.03). From each well three separate 16-bit images in the basic color (red, green, blue) were created and the open-source software CellProfiler was used for the creation of a composite file. For the image analysis the open-source software Fiji was used. For any mathematical operation during the analysis steps, images were converted into a 32-bit floating point format to avoid pixel saturation.

### 2.10 Measurement of ADSC mitochondrial respiration

For the Mito Stress Test, differentiated ADSC were trypsinized and 5000 cells per well were plated as five technical replicates in complete DMEM (L-glutamine, 10% FBS and penicillin-streptomycin) on a 96 well Seahorse Assay Plate and incubated overnight at 37 °C in a humified 5% CO2 incubator. On the day of the assay, DMEM was replaced with Seahorse Assay Medium pH 7.4 (with 10 mM glucose, 2 mM L-glutamine and 1 mM sodium pyruvate) and placed for one hour at 37 °C in a non-CO2 incubator. Afterwards, the plate was placed in a Seahorse XFe96 Extracellular Flux Analyzer (Agilent Technologies) and sequentially oligomycin (1 µM final concentration), FCCP (2 µM final concentration), and Rotenone/antimycin A (0.5 µM final concentration) were added to analysed for mitochondrial respiration. To normalize Seahorse Assay readouts to cell number, cell numbers were counted using a Cytation 2 imaging reader (BioTek).

### 2.11 Statistics

The statical analyses were performed using GraphPad Prism V 9.4 (GraphPad Software, San Diego California, USA). All data represent mean ± SD, depicted in whisker plots exhibiting the 5–95 percentiles. Statistical significance between two groups was calculated by a two-tailed Student’s t-test. Outliers have been excluded from the analysis using Grubb’s test. P<0.05 was accepted as statistically significant.

## 3 Results

### 3.1 Patients with lipedema show abundant levels of immunosuppressive adipose tissue macrophage populations

**Figure 1:**
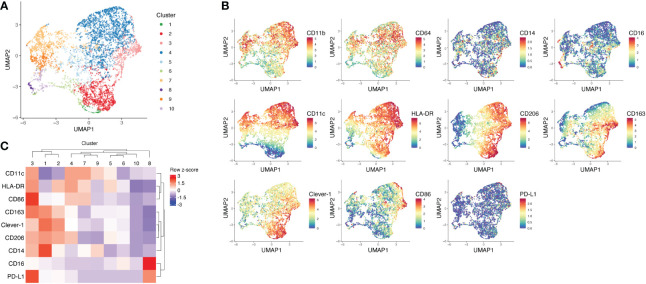
Mass cytometry analysis of SVF macrophages of lipedema patients and BMI matched healthy controls. **(A)** UMAP plot and FlowSOM metaclusters of CD11b^+^CD64^+^ myeloid cells (n = 5708) from lipedema patients (n = 5) and healthy controls (n = 5). The SVF samples were pre-gated for viability, singlets and CD45. **(B)** UMAP plots showing arcsinh-transformed expression values of indicated markers on CD11b^+^CD64^+^ cells. **(C)** Heatmap and unsupervised clustering of FlowSOM metaclusters based on median marker expression in each metacluster.

**Figure 2:**
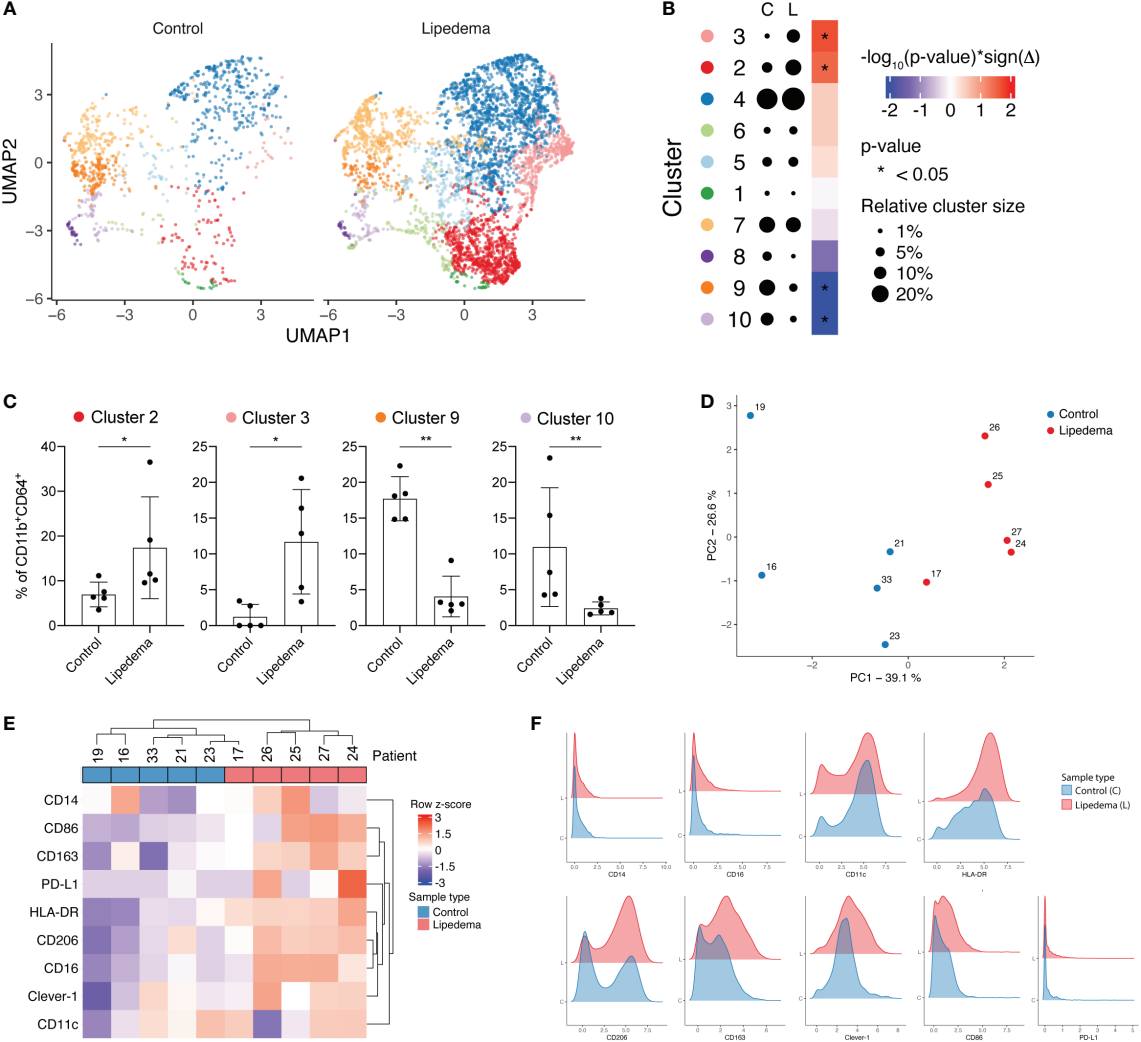
Lipedema patients show higher proportions of immunosuppressive CD206^+^CD163^+^Clever-1^+^ macrophages. **(A)** FlowSOM metaclusters of CD11b^+^CD64^+^ cells displayed on separate UMAP plots of healthy controls (n = 5, 1066 cells) and lipedema patients (n = 5, 4642 cells). **(B)** A dot plot of average FlowSOM metacluster sizes in healthy control and lipedema patient groups. Dot radiuses represent average percentage of cells belonging to each metacluster. Heatmap displays statistical significances (Mann-Whitney U test) with red colour indicating increase and blue colour decrease in comparison to the control group. **(C)** FlowSOM metaclusters that were significantly more or less abundant in lipedema patients. Each dot represents metacluster relative size in a healthy control or a lipedema patient. Mann-Whitney U test. *P < 0.05; **P < 0.01 **(D)** Principal component analysis based on relative FlowSOM metacluster sizes in each individual. **(E)** Unsupervised hierarchical clustering of control and lipedema samples based on median marker expression levels on CD11b^+^CD64^+^ cells. **(F)** Density plots showing the expression of indicated markers on CD11b^+^CD64^+^ cells in control and lipedema group.

To understand how fat accumulation alters adipose tissue macrophages in patients diagnosed with lipedema, we performed CyTOF analysis of CD45^+^CD11b^+^CD64^+^ cells of the SVF and compared their phenotype with SVF macrophages from BMI and location (site of liposuction) matched healthy donors. We identified 10 different metaclusters of phenotypically different macrophages, which displayed varying expression levels of pro-inflammatory markers (CD11c, CD86 and HLA-DR) and immunosuppressive markers (CD206, CD163, Clever-1) (**Figures 1A–C**). Patients with lipedema showed a significant increase in the abundance of immunosuppressive macrophage populations in cluster 2 and cluster 3 (**Figures 2A–C**). Cluster 3 had characteristics of a mixed phenotype with high expression of both inflammatory (CD86) and immunosuppressive markers (CD206, CD163) and high PD-L1 (**Figure 1C**). Principal component analysis (PCA) performed on the relative levels of macrophage metaclusters showed clear separation of patients with lipedema compared to control samples (**Figure 2D**). Also, unsupervised clustering of lipedema and control samples based on median marker expression on CD11b^+^CD64^+^ cells similarly grouped 4 out 5 lipedema patients together (**Figure 2E**). When comparing the marker expression profiles of CD11b^+^CD64^+^ cells between lipedema patients and the control group, lipedema patients showed higher levels of CD163, Clever-1, HLA-DR and CD86, and a higher proportion of CD206 expressing cells (**Figures 2E, F**). Altogether, these data suggest that lipedema induces a shift in adipose tissue macrophages towards a more immunosuppressive (M2) state.

### 3.2 RNA Sequencing of CD11b+ cells shows increased expression of M2 markers

**Figure 3:**
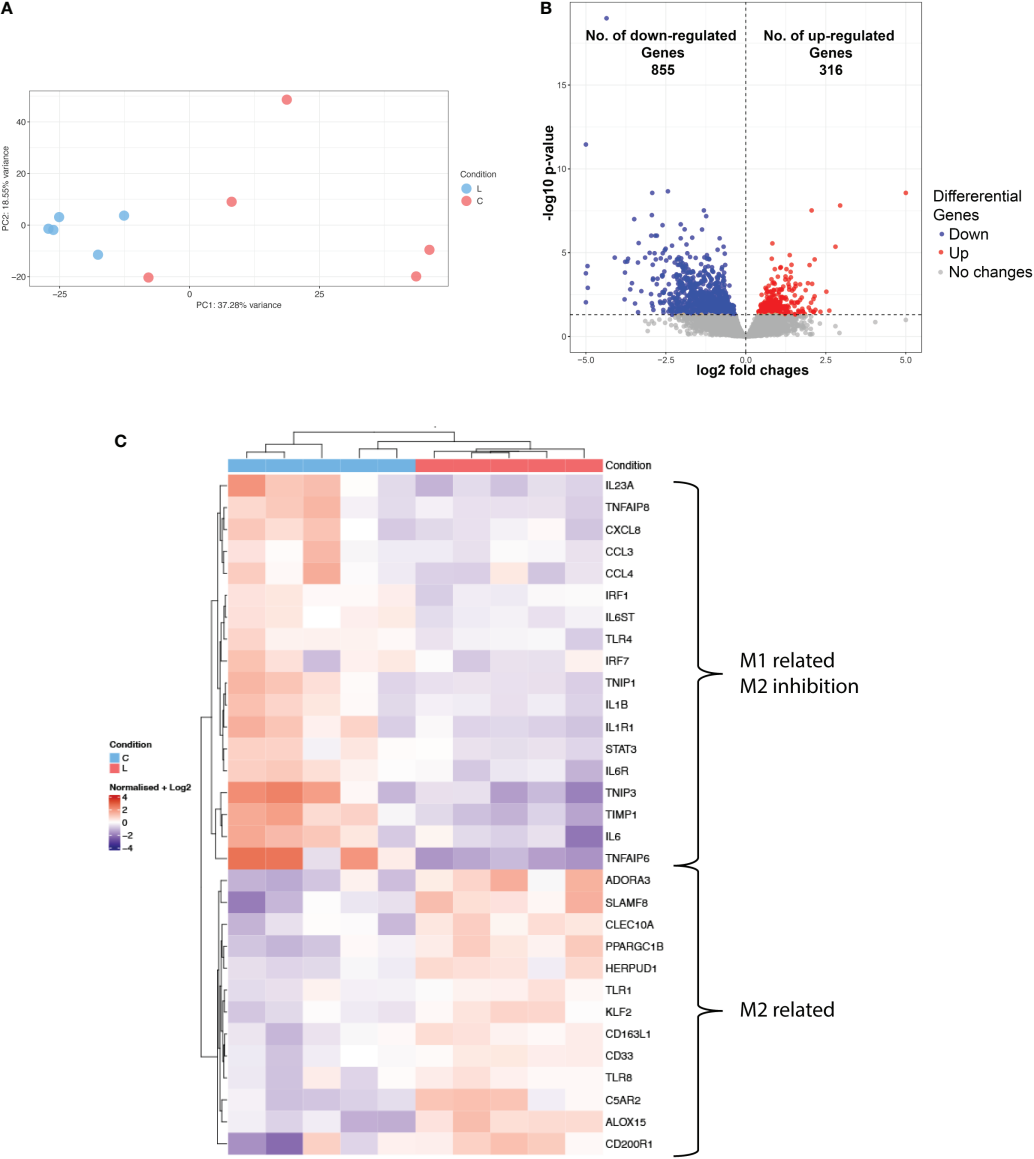
Transcriptional profiling of CD11b+ of lipedema and control patients. **(A)** Principal component analysis of RNA-seq data show the characteristics of samples according to gene expression **(B)** Volcano plot of significantly differentially expressed genes (adj. p < 0.05) **(C)** Heatmap of hierarchical clustering showing expression patterns of selected differentially expressed macrophage-associated genes. Log2 normalized expression value: red indicates upregulation and blue indicates downregulation (N(L) = 5; N(C) = 5).

As the phenotypic characterization indicated a distinct immunosuppressive immune environment in lipedema, we next sought to evaluate the transcriptomic profile of the myeloid compartment and molecular pathways involved. For that purpose, CD11b+ cells were sorted from the SVF from 5 control and 5 lipedema patients and processed for sequencing. Principal component analysis showed a clear separation between the CD11b RNA expression profile of lipedema and control samples (**Figure 3A**) where 1171 differentially regulated genes were identified (**Figure 3B**). Among those genes we mapped the M1 and M2 related genes (**Figure 3C**). Genes that were associated with M2 macrophage phenotype were upregulated, such as A3 adenosine receptor (*ADORA3*), which is involved in survival of anti-inflammatory monocytes (19); genes which were induced upon stimulation of IL4, IL10 and/or IL13 in M2 macrophages such as *CLEC10A* (CD301) and *PPARGC1B* (20), *TLR1* and *TLR8* (21). *ALOX15* (22), CD200 receptor (*CD200R*) (23) and genes, which promote differentiation of the M2 part of the activation spectrum: *HERPUD1* (24), *CD163-L1* (25), *KLF2* (26) and *TIMP-1* (27). On the other hand, typical M1 markers were significantly downregulated: *IL1b*, *IL6*, *IL23a*, *IL1R1* (28) and genes that inhibit M2 macrophage polarization showed reduced expression, such as *TNFAIP8* (29), *TLR4* (30) and *IRF-1* (31).

Hierarchical clustering of the top differentially expressed genes showed distinct subsets of lipedema and non-lipedema genes with gene ontology annotation and pathway enrichment analysis highlighting cytokine-mediated signaling activity, interleukin-1 receptor activity, extracellular matrix organization and regulation of androgen receptor signaling (**Supplementary Figures 2A, B**). A detailed enrichment (Enrichr) analysis of cluster 3, which was upregulated in lipedema, identified the MAP kinase signaling pathway and the PIK3 pathway (**Supplementary Figure 2C**). Both pathways are essential drivers of the polarization of macrophages towards the M2 phenotype (32).

### 3.3 Increased CD163+ cell infiltration and CD163 expression levels in human skin in lipedema

**Figure 4:**
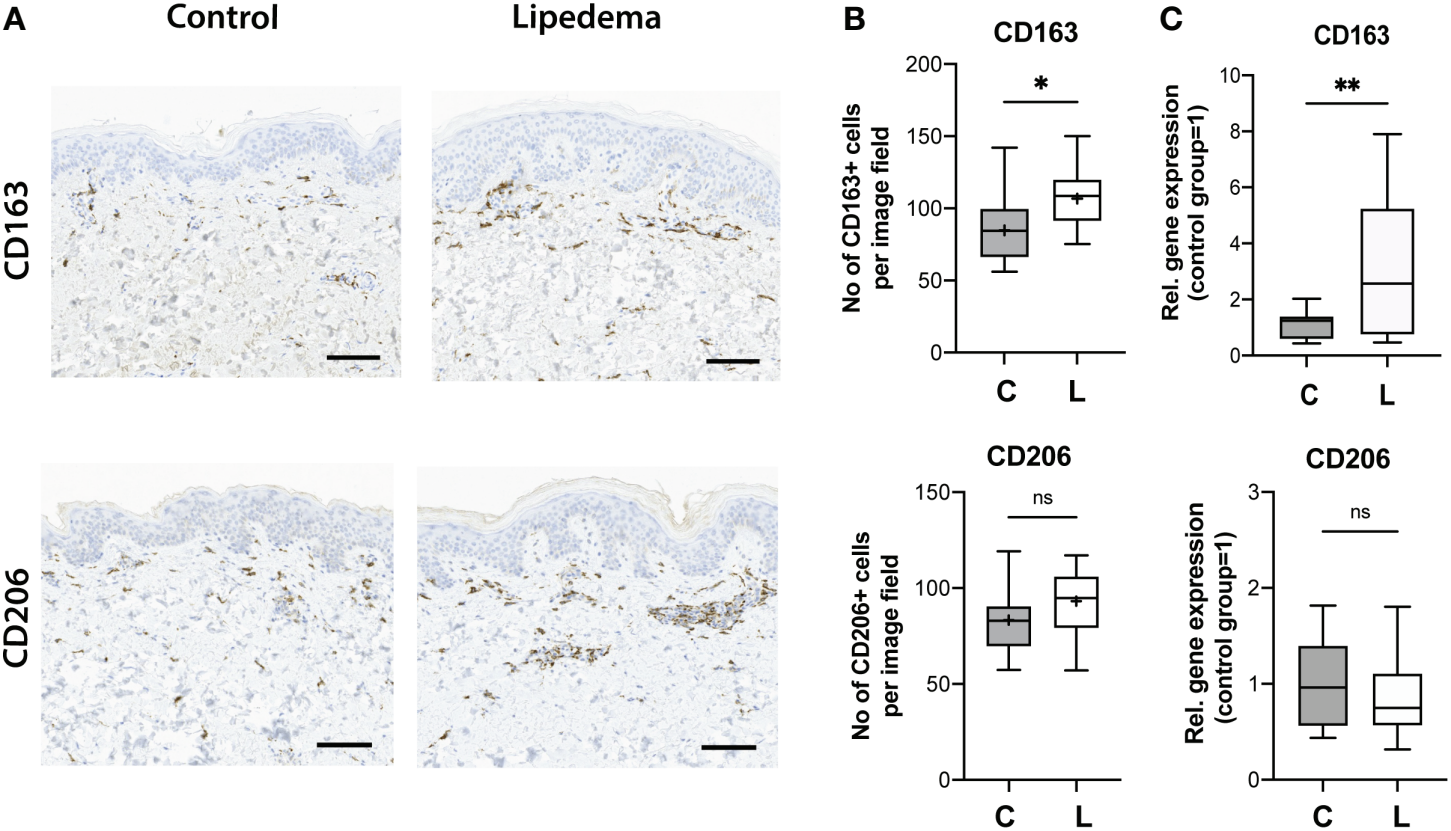
Increased CD163+ cell infiltration and expression levels in human skin in lipedema. **(A)** The immune cell infiltrate was evaluated on paraffin-embedded skin tissue sections. **(B)** Quantification of the CD163+ and CD206+ cells revealed increased infiltration of CD163 + cells in skin tissue. N(C): 14, N(L): 15 **(C)** The evaluation of the CD163 and CD206 expression in fat tissue. N(C): 14, N(L): 15 patients. Scale bar: 100 μm. Asterisks indicate statistical significance in comparison to the control *P < 0.05, **P < 0.01 (two-tailed Student’s t-test). ns, not significant.

The phenotypic characterization of the SVF revealed a distinct immunosuppressive macrophage component, with increased numbers of CD163+ and CD206+ cells. Thus, we next attempted to further confirm these findings in human skin sections from lipedema and control patients. Paraffin-embedded skin sections were used and stained for CD163 and CD206. While only a trend towards increased presence of CD206+ cells (control [C]: 83.3 ± 16.9 cells/field versus lipedema [L] 93.20 ± 16.4 cells/field) was detected without reaching statistical significance, a significant increase of CD163+ cells in the lipedema tissue versus the control was clearly noted (P=0.010) (control [C]: 84.7± 24.6 cells/field versus lipedema [L] 106.8 ± 19.5 cells/field. These findings were further confirmed by evaluating the CD163 (2,58-fold upregulation) and CD206 expression levels, which showed analogous results (**Figure 4**).

### 3.4 IPI-549 normalizes the CD163 expression in the lipedema SVF to the expression level of control group

The results obtained in the study point clearly towards a central role of CD163+ immunosuppressive M2 macrophages in lipedema. CD163+ M2 macrophages are a driving factor in tumor growth, angiogenesis and metastasis (33, 34) and the repolarization towards M1 macrophages presents a novel approach in oncologic treatment (35). IPI-549 is a selective PI3Kγ inhibitor, which switches M2 macrophages towards an M1-like phenotype and results in a downregulation of CD163 (36).

**Figure 5:**
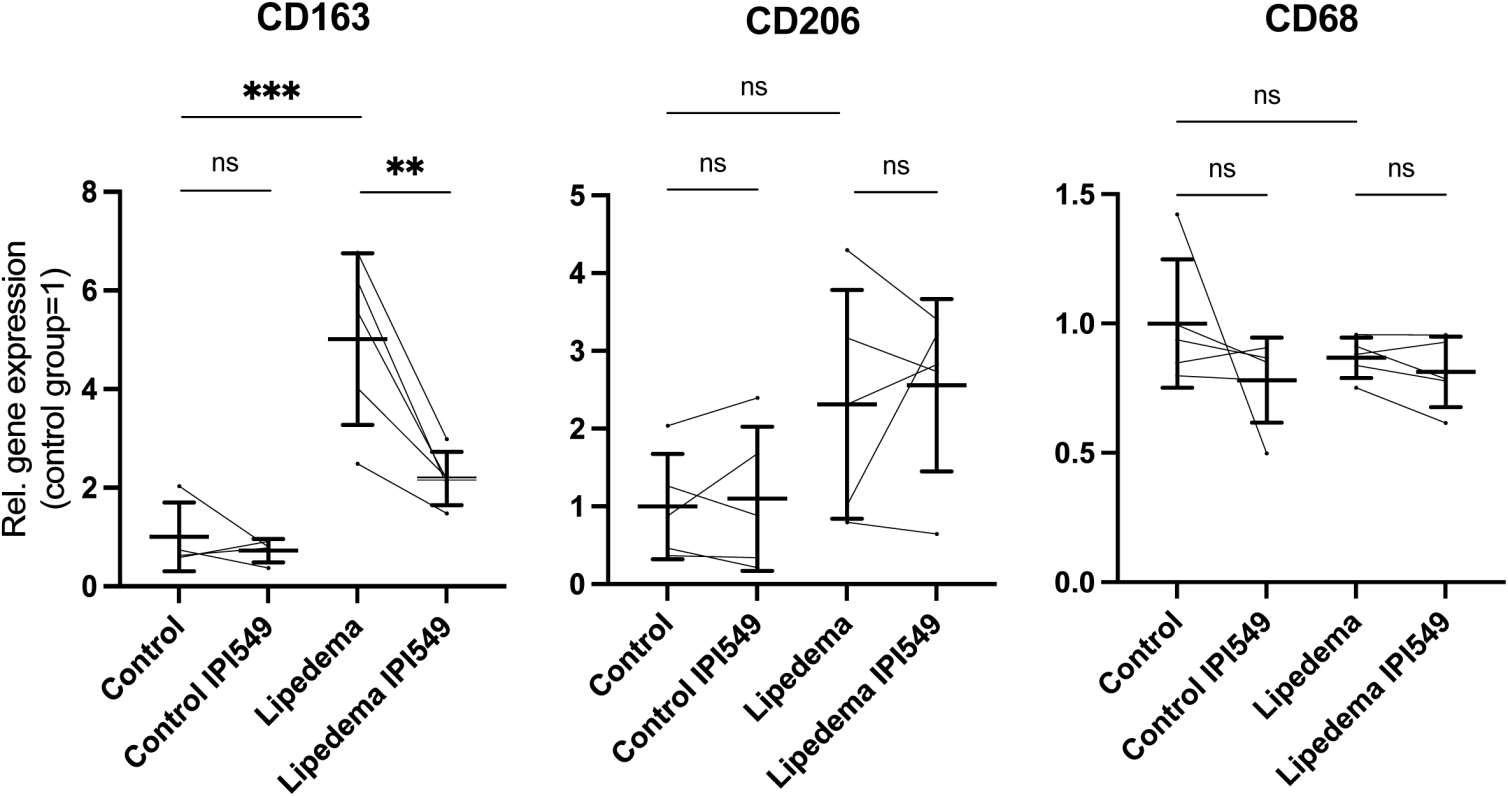
IPI-549 reduces CD163 expression in the lipedema SVF. Evaluation of the gene expression levels of CD163, CD206 and CD68 of IPI-549 treated and untreated SVF of lipedema and control patients. For the comparison between the treated and untreated expression of the same patient a paired Student’s t-test was used, for the comparison between untreated lipedema to untreated control an unpaired two-tailed Student’s t-test was used, N(C): 5, N(L): 5, Asterisks indicate statistical significance in comparison to the control **P < 0.01, ***P < 0.001. ns, not significant.

We treated the SVF from 5 lipedema and 5 control with 100 nM IPI-549 *in vitro* and analyzed the expression of CD163, CD206 and CD68. Firstly, we could confirm our previous results by observing a significant 5-fold upregulation of CD163 in the lipedema SVF compared to the control SVF. Upon treatment with IPI-549, the expression of CD163 in lipedema SVF was significantly reduced, nearly to the level of expression in the control group. The expression levels of CD206 and CD68 remained unaltered by IPI-549 treatment (**Figure 5**).

### 3.5 Conditioned medium from IPI-549 treated lipedema SVF normalizes adipose-derived stem cell differentiation in lipedema

**Figure 6:**
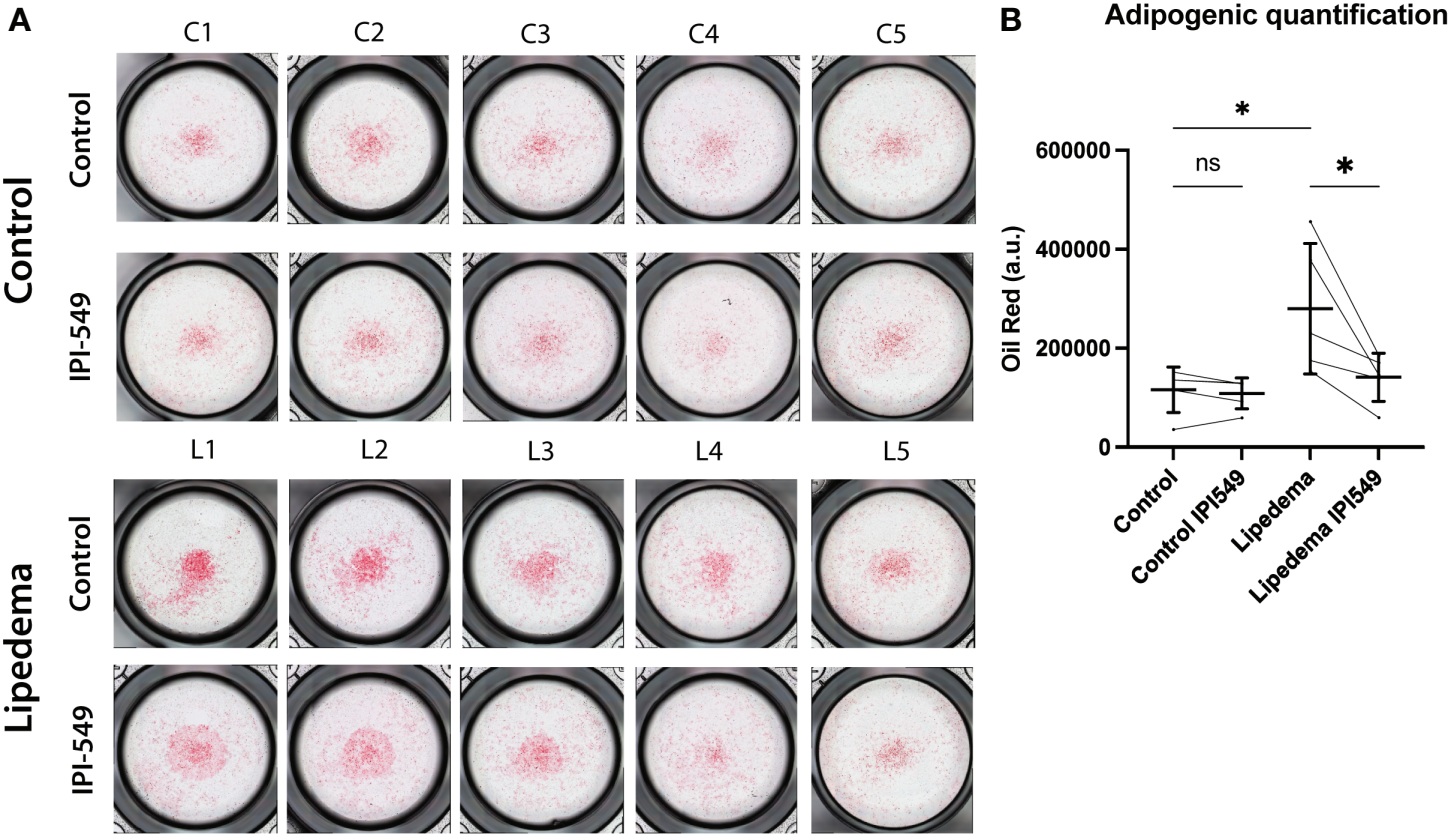
Conditioned medium from IPI-549 treated SVF normalizes adipose-derived stem cell differentiation in lipedema. **(A)** Representative pictures of Oil Red O stained ADSCs which were differentiated with condition medium from IPI-549 treated and untreated SVF of lipedema and control patients. **(B)** Quantification of the Oil Red staining. For the comparison between the treated and untreated sample of the same patient a paired T-test was used, for the comparison of the untreated lipedema to untreated control an unpaired T-test was used, N(C): 5, N(L): 5, Asterisks indicate statistical significance in comparison to the control *P < 0.05. ns, not significant.

Macrophages are known to modulate adipose tissue differentiation and metabolism. To evaluate the functional role of the predominant CD163+ cell population in lipedema we used conditioned medium from lipedema and control SVF, including a treatment with 100 nM IPI-549. The conditioned medium of the treated and untreated groups (from both lipedema and control patients) was used to differentiate adipose derived stem cells (ADSCs) and thus assess the potential contribution of the CD163+ cell population in inducing the formation of adipose tissue. After 7 days of differentiation using the condition medium, a remarkable increase of lipid content was observed in the ADSCs treated with lipedema conditioned medium compared to conditioned medium from control patients, indicating the induction of adipose tissue formation. Surprisingly, the addition of IPI-549, which reduces CD163, resulted in a significant decrease and normalization of the lipid accumulation down to the level of the control SVF. (**Figure 6**) The control (no SVF cells) group did not show a difference between IPI-549 and untreated group, which suggests that IPI-549 does not directly influence the differentiation of the ADSCs (**Supplementary Figure 4**).

**Figure 7:**
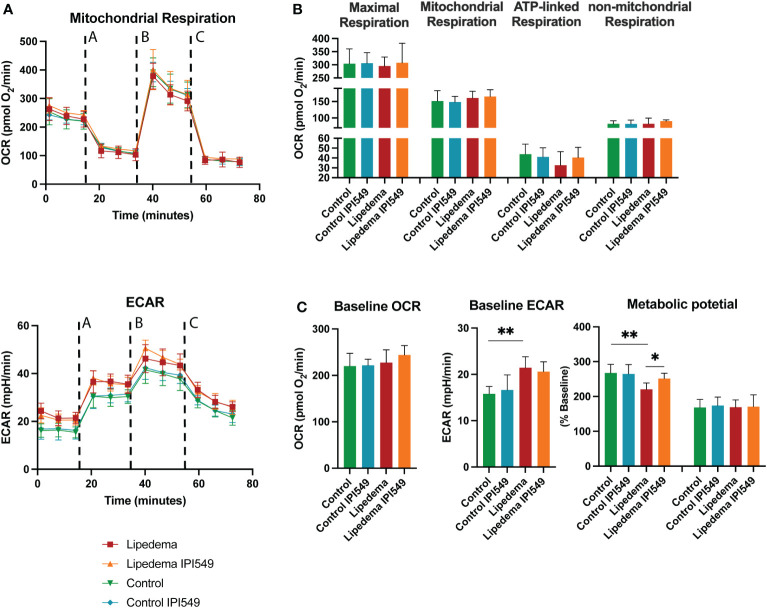
Oxygen consumption rates of differentiated ADSCs with conditioned medium of IPI-549 treated and untreated SVF of lipedema and control patients. **(A)** Evaluation of the oxygen consumption rate (OCR) and extracellular acidification rate (ECAR) changes in response to mitochondrial respiratory function stress test using sequential applications of A = oligomycin, B = FCCP, C = Antimycin A & Rotenone **(B)** No significant alterations were observed in mitochondrial respiration, maximal respiration, ATP-linked respiration nor non-mitochondrial respiration, **(C)** Evaluation of the metabolic phenotype showed alterations of the ECAR baseline and ECAR metabolic potential of ADSCs differentiated with lipedema conditioned medium versus control. The treatment with IPI-549 normalized the lipedema ECAR metabolic potential to the control level. N(C) = 5, N(L) = 5. For the comparison between the treated and untreated sample of the same patient a paired T-test was used, for the comparison of the untreated lipedema to untreated control an unpaired T-test was used. OCR and ECAR values are normalized to cell numbers. *P < 0.05; **P < 0.01.

It is well established that macrophages modulate energy metabolism of adipose tissue in an activation-dependent paracrine way. Therefore, the mitochondrial activity of the differentiated ADSC was evaluated using mitochondrial stress test using ana an Agilent Seahorse XFe96 Analyzer. Basic parameters such as the basal respiration, the ATP-linked respiration (calculated after oligomycin administration) and the non-mitochondrial respiration (after Antimycin A & Rotenone administration) showed no alterations. In order to determine the maximal possible oxygen consumption, the uncoupler carbonyl cyanide-4-(trifluoromethoxy)phenylhydrazone (FCCP) was administered, which also showed no difference. Nevertheless, conditioned medium from lipedema SVFs (without treatment) showed a tendency towards a downregulation of the ATP-linked respiration, in line with previous results demonstrating a decrease of ATP-linked respiration due to CD163+ macrophages (37) (**Figures 7A, B**).

In the next step, we evaluated the oxygen consumption rate (OCR) and the extracellular acidification rate (ECAR) under baseline and stressed conditions (in the presence of oligomycin and FCCP) and calculated metabolic potential ((stressed OCR or ECAR/baseline OCR or ECAR) × 100%) (**Figure 7C**). While the baseline and metabolic potential of the OCR rate remained unaltered, the ECAR baseline of differentiated ADSCs under lipedema conditioned medium was significantly increased compared to the control. Furthermore, the ECAR metabolic potential of differentiated ADSCs under lipedema conditioned medium was reduced compared to the control. The treatment with IPI-549 normalized the metabolic potential to the control level.

## 4 Discussion

In this study, we show that lipedema is characterized by the presence of a distinct immunosuppressive macrophage infiltrate, which decisively influences the adipogenic differentiation evaluated by the lipid accumulation in adipose-derived stem cells. Importantly, the repolarization of the lipedema macrophages was able to normalize adipogenic differentiation and ECAR metabolic potential to the level of the control group, thus limiting the formation of new adipocytes from precursor cells.

Adipose tissue macrophages are shown to play an important role in obesity-associated inflammation and metabolic diseases (38). Macrophages can modulate the energy metabolism and adipocyte mitochondrial function (39). In adipose tissue of obese people, an increased number of pro-inflammatory M1 macrophages is present (6), but in the case of weight reduction, the M1/M2 ratio switches towards an M2 phenotype (6, 40). Over the last decade, several research groups, including our lab, have shown that macrophage infiltration is a hallmark of lipedema as well (5, 41–43).

Here, we analyzed in detail the phenotype of the adipose tissue SVF-derived macrophages of lipedema and BMI- and gender-matched healthy patients using CyTOF. Additionally, RNA sequencing of the CD11b+ cell compartment of lipedema and healthy patients was conducted to elucidate the molecular pathways underlying this distinct immunological niche. The CyTOF analysis revealed that in lipedema, significantly more CD206+CD163+Clever-1+ immunosuppressive M2 macrophages were present, which further confirmed the increased CD163 expression previously observed in lipedema (5). The increase of M1 markers such as HLA-DR and CD68 is explained because HLA-DR and CD86 are present on cells from cluster 2 and 3, where typical M2 marker such as CD206, Clever-1 and our dominant marker CD163 are highly expressed. We obtained similar results from the RNA sequencing of the CD11b+ compartment, where we found an extensive gene expression pattern that supports an immunosuppressive M2 macrophage phenotype. Genes that were associated with the M2 phenotype, such as *CD301*, *CD163L1*, *CD200R1*, *PPARGC1B* and the A3 adenosine receptor were upregulated, while M1 markers such as *IL1b*, *IL6*, *IL23a*, *IL1R1* were less expressed. These changes in gene expression are involved in important functional signaling pathways in macrophages. The proinflammatory interleukin-1 pathway has been shown to be the most prominent downregulated pathway in lipedema macrophages and is accompanied by an altered interleukin-6 pathway, which is responsible for the alternative activation of macrophages (44). What is more, the G coupled receptor A3 adenosine receptor (19) and its downstream pathways are both involved in the regulation of the anti- inflammatory response and upregulated in lipedema CD11b+ cells. In line with these pathways the gene ontology annotation and pathway enrichment analysis of our data identified two clusters, which were associated with immunosuppression. We underpin these results, which indicate strong polarization towards the M2 macrophage phenotype, with an additional immunohistological evaluation of CD163+ cells in the skin tissue of lipedema patients, as well as an expression analysis of CD163+ in fat tissue of lipedema patients, which further confirm the previous findings. CD163+ macrophages are known to be linked with anti-inflammatory functions due to stimulated expression by anti-inflammatory cytokines and their ability to produce anti-inflammatory heme metabolites after CD163-mediated hemoglobin scavenging (45, 46). An enhanced CD163 expression is associated with various inflammatory diseases such as proliferative diabetic retinopathy, systemic lupus erythematosus gestational diabetes mellitus, ulcerative colitis, celiac disease, asthma, lupus nephritis, and rheumatoid arthritis and Crohn’s disease, to name a few (47). Nevertheless, CD163 is also increased in tumor-associated macrophages, a class of inflammatory cells in the microenvironment that is immunosuppressive and supports tumor growth, angiogenesis, and metastasis (33, 34). Additionally, the macrophage infiltration is closely correlated to the prognosis of tumors. The highest levels of CD163+ TAM is found to correlate with the shortest five-year relative survival rates in pancreas, lung and gallbladder cancers (48).

Activated M2 macrophages present the major macrophage population with anti-inflammatory properties in the adipose tissue of lean animals, and they can inhibit adipocyte progenitor proliferation *via* the CD206/TGF-β signaling to modulate systemic glucose homeostasis (49). Recent studies have investigated the effect of macrophage polarization on the differentiation potential of adipose-derived stem cells/preadipocytes. Ma et al. reported that macrophage-derived supernatants inhibit adipogenic differentiation of ADSCs. In particular the M1-macrophage-derived supernatant has a strong inhibitory effect *via* the secretion of TNFa and IL1b (50). Importantly, the expression of *IL1b*, the *IL1R1* and other IL1b related genes were downregulated in the RNA sequencing in our lipedema samples. Yi et al. showed in an *in vitro* assay that M2-polarized macrophages promote adipogenic differentiation of 3T3-L1 cells (51). The adipogenic differentiation seems to be an important factor in the pathophysiology of lipedema (4). However, the current research has focused on the adipogenic potential of lipedema-derived stem cells, but the influence of the immune cell infiltration on adipogenic differentiation in lipedema has not been examined so far. In this study, we could show that SVF-conditioned medium from lipedema patients, where higher numbers of CD163+ cells were present, showed enhanced adipogenic differentiation. To evaluate the potential functional role of the CD163+ cells, we used the selective PI3Kγ inhibitor IPI-549 to reduce CD163 expression on macrophages, converting them to classically activated M1 macrophages. Surprisingly, the differentiation of adipose tissue-derived stem cells with conditioned medium from IPI-549 treated SVF resulted in a significant reduction and normalization of adipose-derived stem cell differentiation evaluated by a decreased accumulation of lipids and normalization of the ECAR metabolic potential in lipedema versus control SVF. However, the increased ECAR baseline in lipedema samples was surprising, because Keuper et al. reported an ECAR decrease during adipogenic differentiation (52). These contradicting results could be explained with a higher release of fatty acids from more lipid-loaded adipocyte that potentially decrease the pH.

The PI3Kγ pathway in macrophages is important for the resolution of inflammation and immunosuppression (32) and specific inhibitors of PI3Kγ are of major interest in combined anti-tumoral therapies (53, 54). IPI-549 is highly specific toward PI3Kγ (53), and a recent study has shown that it leads to almost complete downregulation of CD163 in macrophages (36). Furthermore, it is orally available, displays a good safety profile and is currently evaluated in 4 mid-stage clinical studies in cancer treatment, which are crucial parameters for a quick transition into clinical research.

CD163 has not only potential as a therapeutic target but as a biomarker as well. Upon activation of ADAM17, CD163 is cleaved from the cell surface and forms a soluble CD163 (sCD163), which can be detected in plasma and several tissue fluids (55). sCD163 serves as a marker of macrophage activation and is upregulated in several diseases, such as lupus nephritis (56), multiple sclerosis (57) and proliferative diabetic retinopathy (58). We previously evaluated the serum inflammatory cytokine and chemokine profiles of lipedema patients and controls with a multiplex immunoassay containing sCD163, but did not detect any alterations in their sCD163 levels (9). It is important to mention though, that the sCD163 was evaluated in a relatively low number of patients. Therefore, sCD163 should be the subject of future, larger studies to evaluate its potential as a serum biomarker in lipedema.

One limitation of this study is the relatively low patient number, which is counterbalanced by pursuing a detailed analysis using a variety of complementary methods and the use of anatomically matching samples. Among those, the bulk RNA sequencing was performed on CD11b+ cells, which include not only macrophages but also monocytes, mast cells, neutrophils, NK cells, and subsets of B lymphocytes. While our intention was to include valuable information about possible involvement of further myeloid cell populations in lipedema, the major focus was the in-depth evaluation of the macrophage polarization phenotype and assessment of M1/M2 related genes and involved pathways. While our results indicate that IPI-549 inhibits adipogenic differentiation by reducing CD163 expression on M2 macrophages, we cannot exclude possible effects of IPI-549 treatment on other cell types or a shift of cell populations within the stromal vascular fraction. The SVF consists of a wide variety of cells, such as preadipocytes, endothelial cells, stem cells and immune cells, and IPI-549 treatment may have altered their cytokine secretion. However, our approach aimed to simulate the cellular composition of lipedema *in vitro* by treating the complete SVF with IPI-549 instead of a subpopulation.

Furthermore, we focused only on the immune cell infiltration in lipedema and its influence on adipogenic differentiation. Our *in vitro* approach does not take into account that the onset and development of lipedema *in vivo* cannot be reduced to the altered immune cell infiltration alone. Recent studies have found an altered gene expression and metabolism in stem cells/preadipocytes and adipocytes from lipedema patients, which also contribute to the lipedema phenotype (4). The interplay between the immune cell compartment, in particular macrophages, and the other components of the lipedema tissue should be subject of further studies and may lead one step further to the understanding of lipedema pathophysiology.

In short, here we identified CD163+ macrophages to be a distinct hallmark of the immune cell composition in lipedema and repolarization of lipedema macrophages is able to normalize the differentiation of adipose-derived stem cells *in vitro* evaluated by the cellular lipid accumulation. These data open a new chapter in understanding lipedema pathophysiology and may indicate a potential novel treatment approach.

## Data availability statement

The data presented in the study are deposited in the ENA repository, accession number PRJEB57984.

## Ethics statement

The studies involving human participants were reviewed and approved by Swiss ethics (BASEC-Nr.: 2019-00389) Ethical Committee of the University Hospital Goettingen, State of Lower Saxony, Germany (Nr. 23-11-17, accepted on 23. November 2017). The patients/participants provided their written informed consent to participate in this study.

## Author contributions

EG, SW, MH: conceptualization. EG, MH: supervision. SW, EG, MH, JR, RV, PC: formal analysis. EG, PC, GF, AB, PG, NL: sample acquisition. SW, EG, MH, JR, RV, MD: writing and editing. EG, MH, MD: funding acquisition. All authors contributed to the article and approved the submitted version.
