# Supplementary material for: A distinct M2 macrophage infiltrate and transcriptomic profile decisively influence adipocyte differentiation in lipedema
Source: Front Immunol. 2022 Dec 20;13:1004609. doi: 10.3389/fimmu.2022.1004609 (PMC9809281; doi:10.3389/fimmu.2022.1004609)
Supplement: Supplementary file 1 [file DataSheet_1.docx]

Supplementary Material

# Supplementary Figures and Tables

**Suppl. Table 1.** Patient characteristics

|  | Study cohort |
| --- | --- |
| Number of cases | 39 |
| Lipedema patients | 20 |
| Control patients | 19 |
| Gender |  |
| Female | 39 |
| Male | 0 |
| Mean age (in years) |  |
| Lipedema patients | 48.32 ± 12.69 |
| Control patients | 50.17 ± 12.36 |
| Mean BMI (in kg/m^2^) |  |
| Lipedema patients | 28.60 ± 3.95 |
| Control patients | 26.81 ± 4.43 |
| Lipedema Staging |  |
| Stage I | 1 |
| Stage II | 10 |
| Stage III | 9 |
| Stage IV | 0 |

**Suppl. Table 2.** Number of patients used in each type of analysis

|  | Study cohort | Histological & expression Analysis | CyTOF | RNASeq | Conditioned Medium |
| --- | --- | --- | --- | --- | --- |
| Number of cases | 39 | 29 | 10 | 10 | 10 |
| Lipedema patients | 20 | 15 | 5 | 5 | 5 |
| Control patients | 19 | 14 | 5 | 5 | 5 |
| Gender |  |  |  |  |  |
| Female | 39 | 29 | 10 | 10 | 10 |
| Male | 0 | 0 | 0 | 0 | 0 |
| Lipedema Staging |  |  |  |  |  |
| Stage I | 1 | 1 |  |  |  |
| Stage II | 10 | 6 | 3 | 4 | 4 |
| Stage III | 9 | 8 | 2 | 1 | 1 |
| Stage IV | 0 |  |  |  |  |

**Suppl. Table 3. Primer Sequences**

| Primer | Sequence (5’ to 3’) |
| --- | --- |
| B2M forward | TGTGCTCGCGCTACTCTCTCT |
| B2M reverse | CGGATGGATGAAACCCAGACA |
| CD163 forward | ACATAGATCATGCATCTGTCATTTG |
| CD163 reverse | ATTCTCCTTGGAATCTCACTTCTA |
| CD206 forward | TATGGAATAAAGACCCGCTGACCAG |
| CD206 reverse | TGCTCATGAATCTCTGTGATGCTC |
| CD68 forward | CGAGCATCATTCTTTCACCAGCT |
| CD68 reverse | ATGAGAGGCAGCAAGATGGACC |


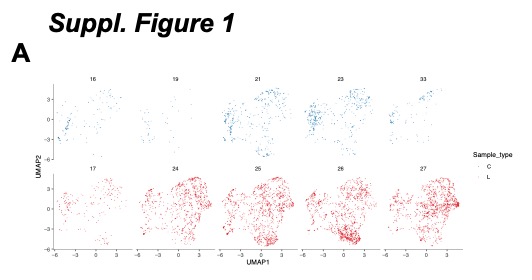


Supplementary Figure 1:

A. Related to Fig. 2A. UMAP plot of each healthy control and lipedema patient displayed separately.

Supplementary Figure 2:

**A.** Hierarchical clustering of the top differentially expressed genes showed distinct subsets of lipedema and non-lipedema genes **B.** with gene ontology annotation and pathway enrichment. **C.** Enrichment (Enrichr) analysis of cluster 3.


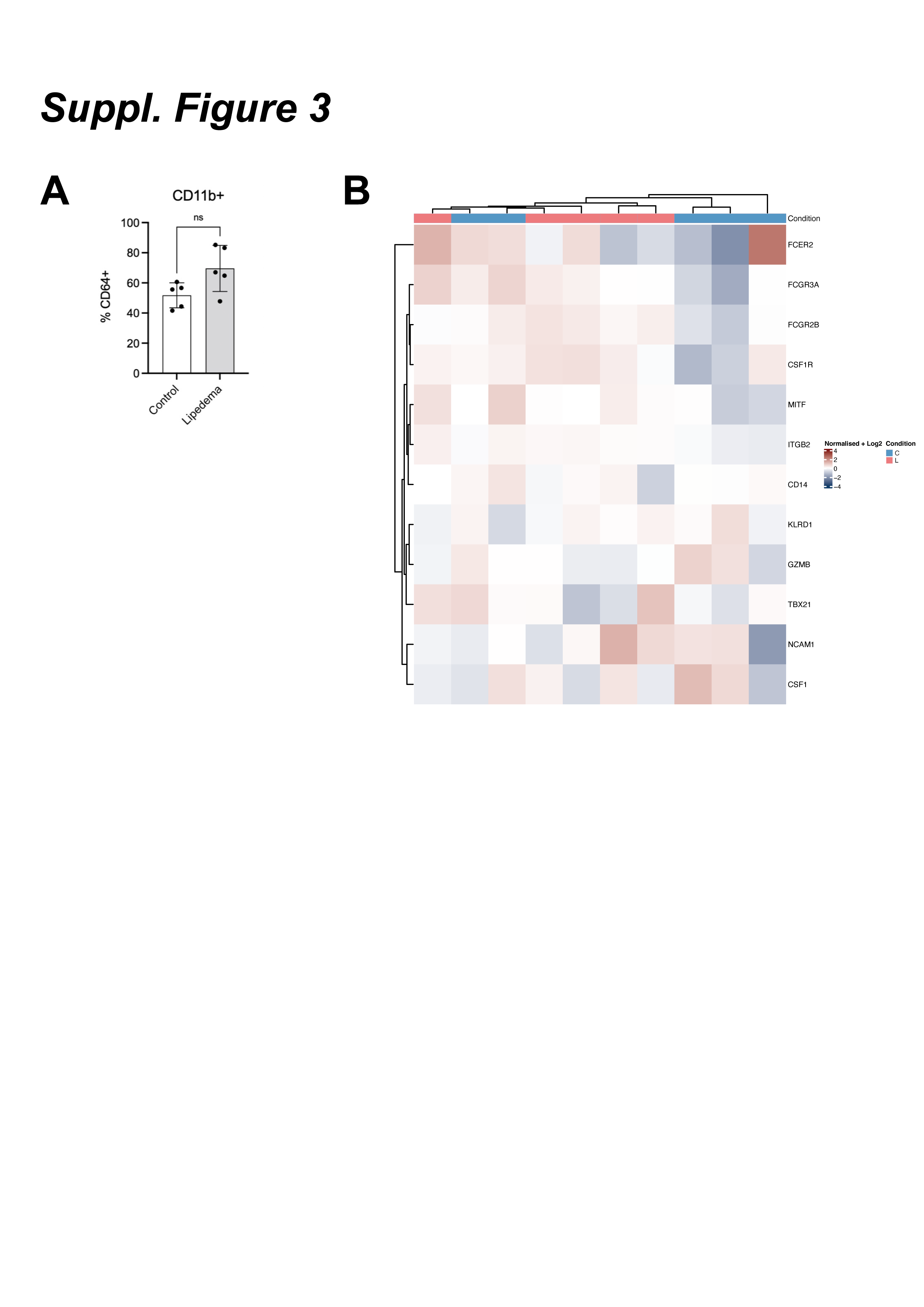


Supplementary Figure 3:

**A.** Quantification of the percentage of Monocytes/Macrophages in CD11b fraction using the CyTOF dataset **B.** Hierarchical clustering of the expressed genes showed similar expression patterns of selected similarly expressed monocyte- (CSF1, CSF1R), neutrophil cell- (FCGR3A, FCGR2B, ITGB2), mast cell- (MITF, FCER2) and NK cell- (NCAM1, KLRD1, TBX21, GZMB) -associated genes.


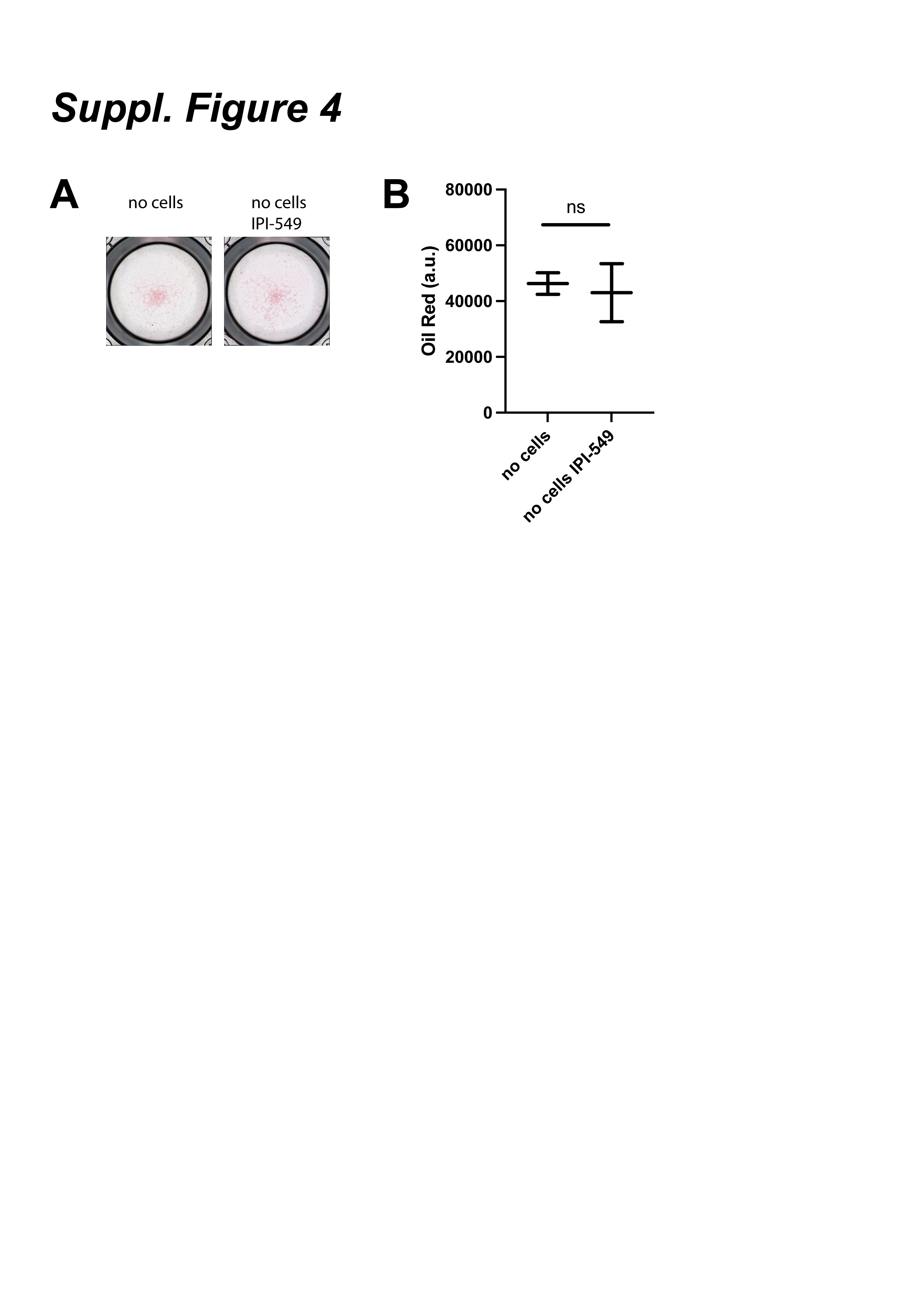


Supplementary Figure 4:

**A.** Representative pictures of Oil Red O stained ADSC which were differentiated in presence or absence of IPI-549. Analog to the main experiments, the IPI-549 containing- and control medium for 72h at 37°C without SVF cells and subsequently used for the differentiation experiment. **B.** Quantification of the Oil Red staining. For the comparison between the treated and untreated sample of the same patient an unpaired T-test was used. (N=3)
